# Post-operative Bleeding Risk in Dental Surgery for Patients on Oral Anticoagulant Therapy: A Meta-analysis of Observational Studies

**DOI:** 10.3389/fphar.2017.00058

**Published:** 2017-02-08

**Authors:** Quan Shi, Juan Xu, Tong Zhang, Bin Zhang, Hongchen Liu

**Affiliations:** Institute of Stomatology, Chinese PLA General HospitalBeijing, China

**Keywords:** oral anticoagulants, dental surgery, post-operative hemorrhage, relative risk, patients, meta-analysis

## Abstract

**Background and Objective:** Minor dental surgery is invasive and hemorrhagic. Thus, in patients treated with anticoagulants, the bleeding risk related to these invasive procedures is concerning. The aim of this meta-analysis is to evaluate this risk by comparing the post-operative bleeding rates of oral anticoagulation treatment (OAT) patients (without interrupted or altered anticoagulant intake) with non-OAT patients.

**Methods:** PubMed, Embase and the Cochrane Library were searched for eligible studies that compared the post-operative (following minor dental surgery) bleeding rates of OAT patients without interrupted or altered therapy with those of non-OAT patients. Relative risk (RR) and 95% confidence interval (CI) were calculated. Subgroup analyses were used to identify the association between the bleeding rate and different dental surgeries or anticoagulants.

**Results:** Thirty two full text articles were assessed for eligibility and 20 studies were excluded according to the selection criteria. Finally, 12 studies and a total of 2102 OAT patients and 2271 non-OAT patients were included. A pooled analysis indicated that the post-operative bleeding risk in OAT patients is higher than that of non-OAT patients (RR: 2.794, 95% CI: 1.722–4.532, *P* = 0.000). The pooled RRs in the dental implant surgery and dental extraction subgroups were 2.136 (95% CI: 0.825–5.531, *P* = 0.118) and 2.003 (95% CI: 0.987–4.063, *P* = 0.054), respectively. As for the different oral anticoagulants, the pooled RR in the subgroup of new oral anticoagulants (NOACs) was 1.603 (95% CI: 0.430–5.980, *P* = 0.482), while the pooled RR in the vitamin K antagonists subgroup was 3.067 (95% CI: 1.838–5.118, *P* = 0.000).

**Conclusion:** Under current evidence, OAT patients were under a higher post-operative bleeding risk than the non-OAT patients following minor dental surgery. For the dental implant surgeries and dental extractions, our study failed to demonstrate a higher risk of bleeding in the OAT patients compared with the non-OAT patients. Besides, The NOACs might be safer than the vitamin K antagonists in dental implant surgery. However, more well-designed studies are required for future research.

## Introduction

Oral anticoagulants (OAs) are commonly used in patients with artificial heart valves, deep vein thrombosis, and pulmonary embolisms to prevent thromboembolic events (Key and Kasthuri, 2010; Andras et al., 2012; Akin et al., 2015). Most of these drugs act as vitamin K antagonists, and their mechanism of action is through inhibition of vitamin K dependent clotting factors (II, VII, IX, X) (Mackman, 2008; Andras et al., 2012). In recent years, new oral anticoagulants (NOACs) have emerged and been used in clinical practice (Ning et al., 2016). Because of the aging populations and high rates of cardiovascular disease in most countries, a large number of patients will be undergoing oral anticoagulant treatment (Madrid and Sanz, 2009; Nocini et al., 2013). However, although oral anticoagulant treatment (OAT) with vitamin K antagonists is effective for prophylaxis of potentially life-threatening thromboembolic events, the risk of post-operative bleeding has been a concern in the medical treatment of patients undergoing OAT compared with non-anticoagulated subjects (Rodríguez-Cabrera et al., 2011; Rowley et al., 2013; Philip et al., 2014).

According to previous reports (Cannon and Dharmar, 2003; Kämmerer et al., 2015), minor dental surgery, which is the common procedure in dental health care and routine outpatient treatment, including tooth extractions, dental implant surgery, mucoperiosteal flaps, periodontal surgical procedures, alveoloplasties, limited oral soft tissue surgery, and augmentation procedures (e.g., elevation of the maxillary sinus). Because most of these dental operations can be invasive and hemorrhagic, concern over the safety and efficacy of the OAT and the accompanying bleeding risks related to these invasive dental procedures is unavoidable (Pototski and Amenabar, 2007; Kosyfaki et al., 2011; Jimson et al., 2015). However, clinical studies evaluating this risk have reported conflicting results. The results of some studies have revealed that the post-operative bleeding rate in patients undergoing OAT was not higher than that in patients not undergoing OAT (Bacci et al., 2010, 2011; Bajkin et al., 2015b). Moreover, using local hemostasis methods, minor dental surgery can be performed safely in anticoagulated outpatients, without any modification of their ongoing anticoagulant therapy. On the contrary, some studies have reported more post-operative bleeding in OAT patients (Eichhorn et al., 2012; Iwabuchi et al., 2014), and OAT may increase the risk of bleeding after these invasive procedures (Hong et al., 2010).

Furthermore, some systemic reviews have discussed these clinical problems, but the conclusions of these reviews were also inconsistent. Two systemic reviews concluded that OAT patients who continue the OA medication do not have a significantly higher risk of post-operative bleeding than non-OAT patients (Madrid and Sanz, 2009; Kämmerer et al., 2015), while Kosyfaki et al. (2011) thought that the OAT patients are at a higher risk for enhanced bleeding tendency compared to the non-OAT patients, when the anticoagulant therapy is continued (Kosyfaki et al., 2011). Moreover, these systemic reviews did not conduct a quantitative analysis to validate their conclusions (Madrid and Sanz, 2009; Kosyfaki et al., 2011; Kämmerer et al., 2015). These conclusions of clinical studies and systematic reviews indicate that whether the post-operative bleeding rate is higher in OAT patients than in the non-OAT patients is need further research.

Because of the special anatomical site of an oral cavity, severe post-surgical bleeding could lead to serious consequences, including life-threatening upper airway obstruction, dysphagia, or post-operative trismus (Evans et al., 2002; Scully and Wolff, 2002; Scully et al., 2007; Kosyfaki et al., 2011). If such events occur, OAT patients would be more at risk than healthy patients, which could discourage OAT patients from seeking future dental treatment (Nematullah et al., 2009). In addition, as patients increasingly tend to maintain their dental health as they get older, dentoalveolar surgery is more often indicated for elderly patients who are taking OAs (Broekema et al., 2014). Therefore, it is of great importance to evaluate the post-operative bleeding risks of OAT patients after minor dental surgery and to take effective hemostatic measures.

Considering that a single study may lack the power to provide reliable conclusion due to the small number of subjects, and lack of quantitative analysis in the available systemic reviews, a meta-analysis is required to quantitatively assess the risk. Therefore, we conducted this meta-analysis by collecting available evidences, which comparing the post-operative (i.e., minor dental surgery) bleeding rates of OAT patients without interruptions or modifications to their therapy with those of non-OAT patients, to evaluate the post-operative bleeding risk of OAT. These results will provide clinicians with better evidence-based evaluations and recommendations.

## Materials and Methods

### Search Strategy and Selection Criteria

In this meta-analysis, we included clinical studies that compared the post-operative bleeding rates (following minor dental surgery) of OAT patients without interrupted or altered therapy and non-OAT patients. Studies with available data that could be extracted were also included. The post-operative bleeding events include: slight bleeding and moderate bleeding, and severe hemorrhaging. The exclusion criteria were (1) animal studies or *in vitro* studies, (2) reviews, case reports or comments, (3) studies without available data that could be extracted, and (4) studies with patients who were also being treated with antiplatelet drugs or undergoing major surgery.

We searched PubMed, Embase for related studies published from January 1985 to December 2016, and the language was restricted to English. Then, we searched the Cochrane Library, without restrictions. The combination of the following keywords was used: “oral anticoagulant,” “oral anticoagulation treatment (OAT),” “bleeding,” and “dental surgery.” Additional studies were identified by manual searches of the reference lists of the related articles and reviews. These results were independently assessed by two reviewers (SQ and XJ), and any disagreement was resolved through discussion with a third reviewer (LHC).

Briefly, based on the inclusion criteria, the studies were selected as follows. First, after eliminating duplicate articles, irrelevant records were excluded by reading the titles and abstracts. Then, full-texts of the potential studies were scanned, and only the studies meeting the inclusion criteria were ultimately included in our meta-analysis.

### Data Extraction and Quality Assessment

The following information was extracted from each included study: the study ID (first author and year of publication), study design, type of dental surgery, characteristics of the subjects (including the number of patients in each group, age range, sex, oral anticoagulant therapy in the OAT group, and number of patients with post-operative bleeding), hemostasis protocol, and follow-up time, as well as a brief conclusion from study. This process was independently performed by two reviewers (SQ and XJ).

The quality assessment was completed by two reviewers (ZT and ZB) using the Newcastle-Ottawa Scale (NOS). In this assessment tool, the study selection, comparability, and outcomes are used to appraise the methodological quality of the included studies, with a maximum of nine points for each study (Wells et al., 2013). NOS scores of 1–3, 4–6, and 7–9 indicated low, moderate, and high study quality, respectively.

### Data Synthesis and Analysis

Comprehensive Meta-Analysis software package (Version 2.0; Biostat) was used to perform the meta-analysis. The relative risk (RR) and 95% confidence interval (CI) were pooled to estimate the risk of post-operative bleeding in the OAT patients compared with the non-OAT patients. Heterogeneity between studies was tested using I^2^ statistics (I^2^ values of 25, 50, and 75% were considered low, moderate, and high, respectively). A fixed effects model was used if the heterogeneity was low; otherwise, a random effects model was used. Sensitivity analysis and cumulative analysis was performed to analysis the stability of the pooled results. Subgroup analyses of the different dental surgeries and different oral anticoagulants were also performed.

## Results

### Study Selection

**FIGURE 1:**
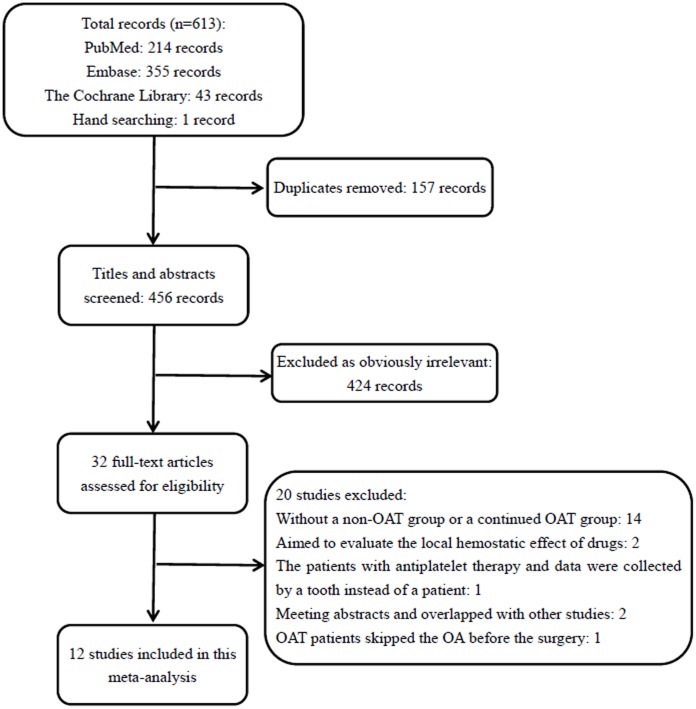
**A flowchart of the retrieved studies**. OAT, oral anticoagulation treatment.

Initially, 613 papers were identified through our search strategy. We reviewed 32 papers in full-text, 14 of which did not have a non-OAT group or a continued OAT group (Al-Mubarak et al., 2006; Hong et al., 2010, 2012; Somma et al., 2010; Morimoto et al., 2011; Bajkin and Todorovic, 2012; Hamid et al., 2012; Healey et al., 2012; Ermer et al., 2013; Svensson et al., 2013; Abdullah and Khalil, 2014; Bajkin et al., 2015a; Dudek et al., 2016; Zirk et al., 2016); two studies aimed to evaluate the local hemostatic effect of drugs (Al-Belasy and Amer, 2003; Cakarer et al., 2013); one study included the patients with antiplatelet therapy, and data was collected by tooth instead by patient (Iwabuchi et al., 2014); two were meeting abstracts and the data overlapped with other studies (Broekema et al., 2013; Febbo et al., 2015); and in the last study, the OAT patients skipped the OA before the dental surgery (Miclotte et al., 2016). Eventually, 12 studies (Campbell et al., 2000; Zanon et al., 2003; Bacci et al., 2010, 2011; Karslı et al., 2011; Eichhorn et al., 2012; Broekema et al., 2014; Bajkin et al., 2015b; Clemm et al., 2016; Febbo et al., 2016; Gómez-Moreno et al., 2016a,b) were included in our meta-analysis. A flow diagram of the study selection process is shown in **Figure 1**.

### Summary of the Included Studies

The publication dates of the 12 included studies ranged from 2000 to 2016. Ten studies (Campbell et al., 2000; Zanon et al., 2003; Bacci et al., 2010, 2011; Karslı et al., 2011; Broekema et al., 2014; Bajkin et al., 2015b; Clemm et al., 2016; Gómez-Moreno et al., 2016a,b) are prospective studies and two are retrospective studies (Eichhorn et al., 2012; Febbo et al., 2016). Four studies (Bacci et al., 2011; Clemm et al., 2016; Gómez-Moreno et al., 2016a,b) researched dental implants in OAT patients, and four studies (Zanon et al., 2003; Bacci et al., 2010; Karslı et al., 2011; Febbo et al., 2016) focused on dental extractions. In the last four studies (Campbell et al., 2000; Eichhorn et al., 2012; Broekema et al., 2014; Bajkin et al., 2015b) the surgeries included dental extraction, apicoectomy, dental implant surgery, limited intraoral soft tissue surgery, and so on. All OAT patients maintained their anticoagulation medication therapies. The value of the international normalized ratio (INR) in the included studies was less than 4.2 (**Table 1**). To stop the intraoperative and post-operative bleeding, all studies adopted local hemostatic measures. These measures and main conclusions of the included studies can be found in the Supplementary Table A1.

**Table 1:** Summary of the included studies.

Study ID	Study design	Dental surgery	Oral anticoagulant therapy	Follow up time	NOS score
Febbo A 2016	Retrospective	Dental extraction	Patients were treated with vitamin warfarin and INR less than 4	Day 1 to day 10	7
Gómez-Moreno G 2016b	Prospective	Dental implant surgery	Patients had been taking dabigatran for over 6 months (150 mg orally every 12 h).	Days 3, 8	8
Gómez-Moreno G 2016a	Prospective	Dental implant surgery	Patients had been treated with rivaroxaban for over 6 month	Days 3, 8	8
Clemm R 2016	Prospective	Dental implant surgery	Patients were treated with vitamin K inhibitors or direct oral anticoagulants.	Day 10	7
Bajkin BV 2015	Prospective	Dental extraction, apicectomy, etc.	Patients were treated with vitamin K antagonists and INR: 2.0–4.2	Days 1, 2, 5, 7	7
Broekema FI 2014	Prospective	Dental extraction, apicectomy, dental implant surgery	Patients were treated with vitamin K antagonists and INR: 1.8–3.5.	1 week	7
Eichhorn W 2012	Retrospective	Osteotomies, apicoectomies, extractions, etc.	Patients were treated with coumarins, INR : 1.2- 4.2	Days 1, 7, 10, 14	5
Karslı ED 2011	Prospective	Dental extraction	Patients on warfarin treatment without interruption, INR < 4.0	48 h	6
Bacci C 2011	Prospective	Dental implant surgery	INR: 1.8–2.98; warfarin therapy >6 months; normal hemoglobin value and platelet count	Days 3, 8	8
Bacci C 2010	Prospective	Dental extraction	Patients were treated with warfarin for at least 3 months, and the therapy was maintained unchanged. INR: 1.8–4	Days 3, 8	8
Zanon E 2003	Prospective	Dental extraction	Patients on warfarin without changed. INR: 1.8–4	Days 3, 8	7
Campbell JH 2000	Prospective	Extraction, alveoloplasty, intraoral soft tissue surgery	Patients continued their anticoagulant regimens. INR:1.2–2.9	Day 1	5

*INR, international normalized ratio*.

The quality assessment results are shown in **Table 1**, using the NOS. Nine studies (Zanon et al., 2003; Bacci et al., 2010, 2011; Broekema et al., 2014; Bajkin et al., 2015b; Clemm et al., 2016; Febbo et al., 2016; Gómez-Moreno et al., 2016a,b) scored more than seven points and were considered to be of high quality. Three studies (Campbell et al., 2000; Karslı et al., 2011; Eichhorn et al., 2012) scored less than six points and were considered to be of moderate quality. No studies were found with low quality.

From these included studies, a total of 2271 non-OAT patients and 2102 OAT patients were studied. Twenty-five of the non-OAT patients presented with post-operative bleeding, and the total bleeding rate was 1.10%. In the OAT group, 91 patients presented with post-operative bleeding, and the total bleeding rate was 4.33%, which was higher than that in the non-OAT group (**Table 2**). Totally, two OAT patients in two studies developed a severe bleeding and needed to be hospitalized (Supplementary Table A1).

**Table 2:** Patient characteristics and post-operative bleeding rates in the included studies.

Study ID	OAT group				Non-OAT group			
	Total number (male/female)	Age (mean ± SD)	Number of bleeding cases	Rate	Total number (male/female)	Age (mean ± SD)	Number of bleeding cases	Rate
Febbo A 2016	439 (278/161)	69.53^a^	9	2.05%	439 (234/205)	65.19 ^a^	0	0%
Gómez-Moreno G 2016b	29 (19/10)	49–71 (66.7 ± 9.15)	2	6.90%	42	NR	2	4.76%
Gómez-Moreno G 2016a	18 (12/6)	46–73 (64.4 ± 7.84)	1	5.56%	39	NR	2	5.13%
Clemm R 2016	46^b^	NR	2	4.35%	447	NR	3	0.67%
Bajkin BV 2015	125 (81/44)	65.6 ± 9.83	7	5.60%	85 (52/33)	64.5 ± 9.8	1	1.18%
Broekema FI 2014	32^b^	NR	3	9.38%	103 (68/35)	21–85	2	1.94%
Eichhorn W 2012	637 (423/214)	26–93 (68 ± 12.1)	47	7.38%	285 (138/147)	23–88 (64 ± 10.3)	2	0.70%
Karslı ED 2011	13 (7/6)	32–69 (46.2 ± 11)	6^c^	46.15%	13 (7/6)	29–61 (40.38 ± 9.23)	3**^c^**	23.08%
Bacci C 2011	52^d^ (39/13)	41–77 (56.2 ± 8.88)	2	3.85%	109	NR	3	2.75%
Bacci C 2010	451 (246/205)	38–89 63.5^a^	7	1.55%	449 (202/247)	35–92 66.4^a^	4	0.89%
Zanon E 2003	250 (59/191)	44–88	4	1.60%	250 (84/166)	42–92	3	1.20%
Campbell JH 2000	12	NR	1	8.33%	10	NR	0	0%
**Total**	**2102**		**91**	**4.33%**	**2271**		**25**	**1.10%**

^a^*Mean age of the patients; ^b^data from the OAT group were a subgroup in the original research; ^c^in this study, the patients who used extra gauze swabs during the first 48 h for bleeding control were counted; ^d^two patients were excluded in the final analysis. NR, not reported*.

### Meta-analysis

#### Primary Outcomes

**FIGURE 2:**
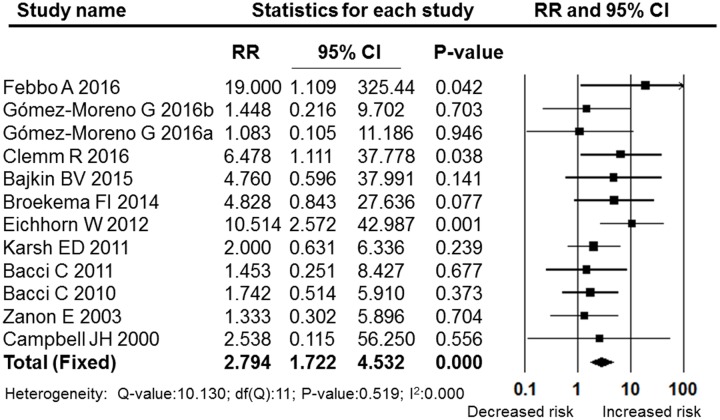
**Forest plot of the post-operative bleeding risk comparison between OAT patients and non-OAT patients**. RR, relative risk; CI, confidence interval.

**FIGURE 3:**
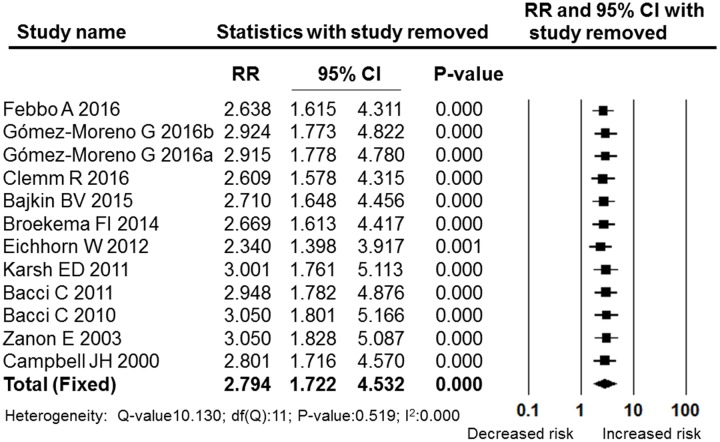
**Forest plot of the sensitivity analysis performed by sequential removing single studies**. RR, relative risk; CI, confidence interval.

**FIGURE 4:**
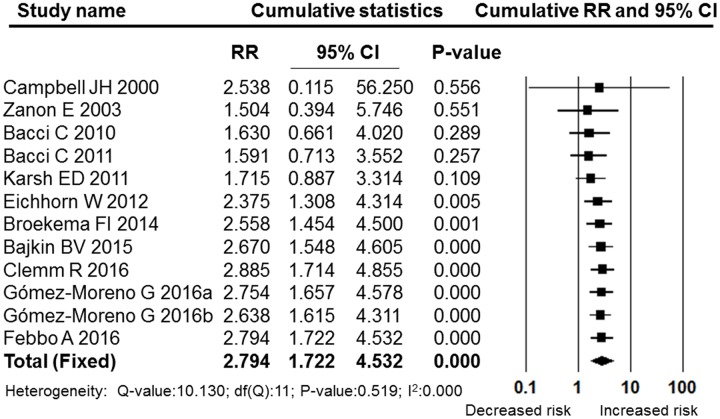
**Forest plot of the cumulative meta-analysis by publication year**. RR, relative risk; CI, confidence interval.

A meta-analysis of the post-operative bleeding rate in the OAT and non-OAT groups was performed. The pooled RR and 95% CI for the OAT patients versus non-OAT patients was 2.794 (95% CI: 1.722–4.532), and this difference was statistically significant (*P* = 0.000, **Figure 2**). The I^2^ value was 0, therefore, heterogeneity between the included studies was low, and a fixed effects model was selected. The sensitivity analysis performed by sequentially removing individual studies revealed stable and statistically significant results (**Figure 3**). Furthermore, a cumulative analysis performed by the order of publication date showed that with overlay of numbers of studies and patients, the results tend to be stable (**Figure 4**)

#### Secondary Outcomes

**FIGURE 5:**
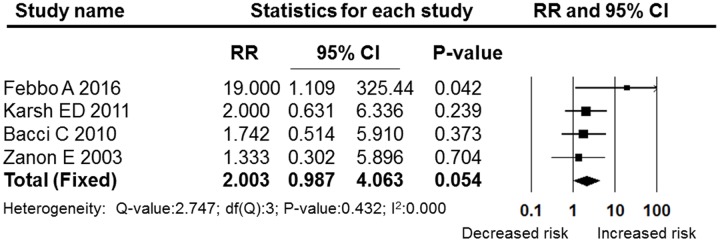
**Forest plot of the post-operative bleeding risk comparison between OAT patients and non-OAT patients in the dental extraction subgroup**. RR, relative risk; CI, confidence interval.

**FIGURE 6:**
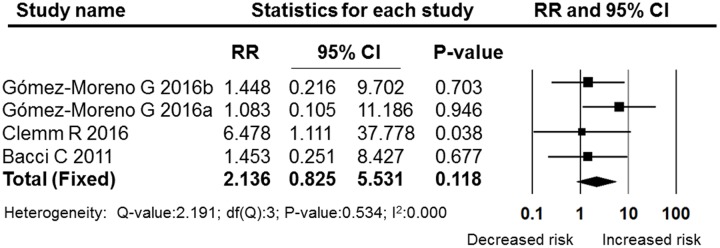
**Forest plot of the post-operative bleeding risk comparison between OAT patients and non-OAT patients in the dental implant subgroup**. RR, relative risk; CI, confidence interval.

**FIGURE 7:**
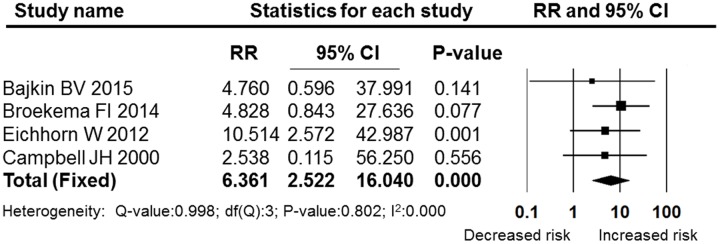
**Forest plot of the post-operative bleeding risk comparison between OAT patients and non-OAT patients in the mixed surgery subgroup**. RR, relative risk; CI, confidence interval.

**Figures 5**–**7** show the subgroup analysis results of the different minor dental surgeries. For dental implant surgery and dental extraction, the pooled RRs were 2.136 (95% CI: 0.825–5.531, fixed effect model, I^2^ = 0, **Figure 6**) and 2.003 (95% CI: 0.987–4.063, fixed effect model, I^2^ = 0, **Figure 5**), respectively. The results indicated no significant difference between the dental implant surgery and dental extraction subgroups (*P* = 0.118 and *P* = 0.054, respectively). The final studies were composed of mixed dental surgeries, and the pooled RR was 6.361 (95% CI: 2.522–16.040, fixed effect model, I^2^ = 0), which indicated a significant difference (*P* = 0.000, **Figure 7**).

**FIGURE 8:**
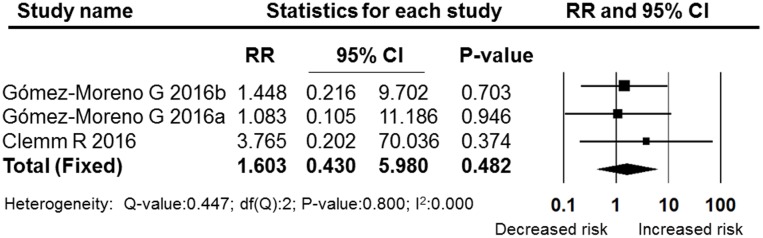
**Forest plot of the post-operative bleeding risk comparison between patients treated with vitamin K antagonists and non-OAT patients**. RR, relative risk; CI, confidence interval.

**FIGURE 9:**
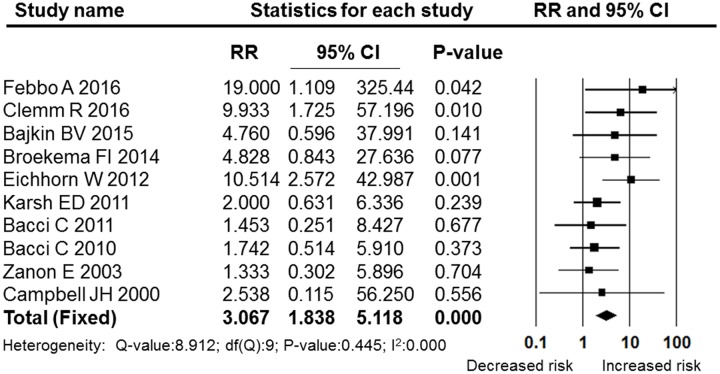
**Forest plot of the post-operative bleeding risk comparison between patients treated with NOACs and non-OAT patients**. RR, relative risk; CI, confidence interval.

The OAT patients in nine studies (Campbell et al., 2000; Zanon et al., 2003; Bacci et al., 2010, 2011; Karslı et al., 2011; Eichhorn et al., 2012; Broekema et al., 2014; Bajkin et al., 2015b; Febbo et al., 2016) were being treated with vitamin K antagonists, and two studies focused on rivaroxaban (Gómez-Moreno et al., 2016a) and dabigatran (Gómez-Moreno et al., 2016b), which belong to the NOACs group. Both of these two types of anticoagulants were extracted from the (Clemm et al., 2016) study through our meta-analysis. In the NOACs subgroup, the pooled RR was 1.603 (95% CI: 0.430–5.980), which failed to show that treatment with NOACs increased the post-operative bleeding rate compared with non-OAT patients (*P* = 0.482, I^2^ = 0, fixed effects model, **Figure 8**). In the subgroup of vitamin K antagonists, the pooled RR was 3.067 (95% CI: 1.838–5.118), which was a significant difference (*P* = 0.000, I^2^ = 0, fixed effect model). These results are shown in **Figure 9**.

## Discussion

The question about discontinued, reducing or maintaining before minor dental surgeries has been a controversial subject. A definitive, standardized protocol for managing minor dental surgery in anticoagulated patients is still lacking (Bacci et al., 2010). It was suggested that OAT patients (INR 2–4) who continued their anticoagulants do not have a significantly higher risk of post-operative bleeding than non-OAT patients (Madrid and Sanz, 2009). However, some reviews concluded that more minor bleedings might occur in OAT patients and that the use of local hemostatic measures, as well as close and extended post-operative monitoring, is vital (Gacina et al., 2006; Kämmerer et al., 2015). Nevertheless, these reviews only had carried on the qualitative description without quantitative analysis. Therefore, it is still disputed whether OAT would increase the post-operative bleeding risk following minor dental surgery, an invasive and hemorrhagic procedure. Our meta-analysis focused on this question, and we performed a quantitative analysis to evaluate the risk by comparing the post-operative bleeding rates of OAT patients without interrupted or altered anticoagulant use and non-OAT patients.

Based on our search strategy and selection criteria, 12 studies (Campbell et al., 2000; Zanon et al., 2003; Bacci et al., 2010, 2011; Karslı et al., 2011; Eichhorn et al., 2012; Broekema et al., 2014; Bajkin et al., 2015b; Clemm et al., 2016; Gómez-Moreno et al., 2016a,b) were included in our meta-analysis. The post-operative bleeding rate in the OAT group (4.32%) was higher than that in the non-OAT group (1.10%). The pooled RR for the OAT patients versus the non-OAT patients was 2.794 (95% CI: 1.722–4.532), and the difference was statistically significant, which indicates that OAT will increase the risk of post-operative bleeding following minor dental surgery. Furthermore, sensitivity analysis performed by sequentially removing individual studies did not change the results. To stop the bleeding, all included studies used local hemostatic methods after the dental surgeries, while only two OAT patients in two studies developed a severe bleeding and needed to be hospitalized (Clemm et al., 2016; Febbo et al., 2016). Evidence from the current analysis suggested that following minor dental surgery, patients who were treated with oral anticoagulants might have a higher post-operative bleeding risk compared with non-OAT patients, which differs from the previous systematic review (Madrid and Sanz, 2009). Fortunately, using careful local hemostatic methods, the bleeding can be stopped effectively.

As previously mentioned, the value of heterogeneity between studies was tested using I^2^ statistics. A fixed effects model was used if the heterogeneity was low; otherwise, a random effects model was used. We know that when the heterogeneity is low, a fixed model will give us a more reliable result. While when the heterogeneity was high, subgroup analysis will be made to explore the source of heterogeneity. Despite the overall heterogeneity was low in this study, we had still made the subgroup analysis. Here are the reasons: first, minor dental surgery is not a single operation and it includes several types of surgeries; OAs are also composed of several kinds of drugs. The most common OAs, vitamin K antagonists and NOACs, have different mechanism of action. Therefore, we want to identify the association between the bleeding rate and different dental surgeries or anticoagulants to give more guidance for clinicians.

Because the category of minor dental surgery includes several types of surgeries, we performed a subgroup analysis to evaluate the post-operative bleeding risk in the different minor dental surgeries in the included studies. Dental implants are generally considered effective and reliable restorations to replace lost teeth and to restore masticatory function (Shi et al., 2016), and they have been increasingly accepted by patients. Four studies (Bacci et al., 2011; Clemm et al., 2016; Gómez-Moreno et al., 2016a,b) focused on dental implant surgery, and the subgroup meta-analysis revealed that the pooled RR was 2.136 (95% CI: 0.825–5.531), with no significant difference (*P* = 0.17). Four studies (Zanon et al., 2003; Bacci et al., 2010; Karslı et al., 2011; Clemm et al., 2016) focused on dental extractions, one of the most common dental surgeries, and the results of the subgroup meta-analysis (pooled RR: 2.003, 95% CI: 0.987–3.063, *P* = 0.054) was similar to the dental implant subgroup meta-analysis. The results might indicate that with strong local hemostatic measures, the post-operative bleeding risk of OAT patients is not higher than that of non-OAT patients. The operations in the other four studies (Campbell et al., 2000; Eichhorn et al., 2012; Broekema et al., 2014; Bajkin et al., 2015b) were mixed (i.e., several dental surgeries). However, the results reveal that the pooled RR was 6.361 (95% CI: 2.522–16.040, *P* = 0.000), indicating that the bleeding risk of the OAT patients was high. Considering the heterogeneity of the surgeries in these three studies, more studies that focus on single surgeries will be needed in the future. Despite the level of evidence about what extent a safe and successful dental treatment in OAT patients is feasible was lacking (Kosyfaki et al., 2011), however, the intraoperative operation must be as atraumatic as possible to reduce post-operative bleeding.

Vitamin K antagonists (e.g., warfarin) are commonly used in patients with artificial heart valves, deep vein thrombosis, etc. (Key and Kasthuri, 2010; Andras et al., 2012; Cocco et al., 2016). According to the guidelines developed at the Academic Centre for Dentistry Amsterdam (ACTA), the INR of the patients taking vitamin K antagonists (which is measured within 24–72 h preoperatively) must be ≤3.5 (Broekema et al., 2014). While other studies have reported that an INR < 4 was a safe range prior to dental surgery (Evans et al., 2002; Bacci et al., 2011; Karslı et al., 2011; Jimson et al., 2015). The INR value was less than 4.2 in nine of the included studies in which the OAT patients were being treated with vitamin K antagonists. The pooled RR in the vitamin K antagonists subgroup analysis was 3.067 (95% CI: 1.838–5.118, *P* = 0.000), indicating that under the current range, the bleeding rate of the patients treated with vitamin K antagonists was higher than that of the non-OAT patients. However, in the subgroup of NOACs, the pooled RR was 1.603 (95% CI: 0.430–5.980, *P* = 0.482). Based on the two opposite pooled analysis results above, we may conclude that The NOACs might be safer than the vitamin K antagonists in minor dental surgery. Furthermore, the patients treated with NOACs in these three studies underwent dental implant surgery, hence the results of the subgroup analysis indicated that the NOACs might be safer than the vitamin K antagonists for dental implant surgery. Our conclusion was similar to that of Gómez-Moreno et al. (2016a), who reported that implant insertion surgery has been made safer and easier, and does not require special monitoring for patients being treated with these new anticoagulants (Gómez-Moreno et al., 2016a). However, considering the limited number of studies in the subgroup of NOACs and the lacking of direct evidence that compared the vitamin K antagonists and NOACs, more studies are required in the future.

To our knowledge, this is the first meta-analysis to estimate the association between OAT and post-operative bleeding following minor dental surgery by quantitative analysis, especially grouped by different dental surgeries and different oral anticoagulants. Not only did we research electronic databases to identify potential interests, but also we manually examined reference lists from relevant studies. The NOS assessment tool was used to evaluate each of the included studies and none of them had low quality. In the meta-analysis and subgroup analyses, the included studies had low heterogeneity, which significantly increased the statistical power of our analysis. Moreover, sensitivity and cumulative analyses were performed to determine the stability of the pooled results. However, our meta-analysis has three limitations. First, although the included studies had low heterogeneity, the types of surgery and the oral anticoagulants used differed. Second, the search was limited to English-language studies, which might have introduced selection bias to this meta-analysis. Third, because of limit information provided by the included studies, we did not explore the bleeding risk of OAT patients by controlling for factor affecting bleeding risk such as INR value.

In summary, the following conclusions were made: (1) The OAT patients had a higher post-operative bleeding risk than the non-OAT patients following minor dental surgery, and the local hemostatic methods effectively stopped the bleeding; (2) For the dental implant surgeries and dental extractions, our study failed to demonstrate a higher risk of bleeding in the OAT patients compared with the non-OAT patients; (3) The NOACs might be safer than the vitamin K antagonists in dental implant surgery. The present meta-analysis will give clinical doctors a better understanding of the risks of post-operative bleeding in OAT patients after minor dental surgery and help patients make rational decisions. Besides, more well-designed studies with adequate controls for confounding factors are required for future research.

## Author Contributions

The sections on literature research, study selection and data extraction were completed by QS and JX; the section on risk of bias evaluation and data analysis were completed by TZ and BZ; QS drafted the manuscript and JX helped to revise the manuscript. HL is the corresponding author, and he undertook the work of designing this meta-analysis, coordinating and helping to draft the manuscript. All authors read and approved the final manuscript.

## Conflict of Interest Statement

The authors declare that the research was conducted in the absence of any commercial or financial relationships that could be construed as a potential conflict of interest.
