# Supplementary material for: Post-operative Bleeding Risk in Dental Surgery for Patients on Oral Anticoagulant Therapy: A Meta-analysis of Observational Studies
Source: Front Pharmacol. 2017 Feb 8;8:58. doi: 10.3389/fphar.2017.00058 (PMC5296357; doi:10.3389/fphar.2017.00058)
Supplement: Supplementary file 1 [file Table_1.DOCX]

Supplementary Material

Postoperative bleeding risk in dental surgery for patients on oral anticoagulant therapy: a meta-analysis of observational studies

Quan Shi^1#^, Juan Xu ^1#^, Tong Zhang^1^, Bin Zhang^1^, Hongchen Liu^1*^

^1^ Institute of Stomatology, Chinese PLA General Hospital, Beijing, China

*** Correspondence:**Hongchen Liu
liuhc301@hotmail.com

^#^ These authors contributed equally to this work.

**Table A1. Summary of the haemostasis protocol and main conclusion in the included studies.**

| **Study ID** | **Definition of bleeding** | **Severe situation or complications** | **Protocol of haemostasis** |  | **Main conclusion** |
| --- | --- | --- | --- | --- | --- |
| Febbo A 2016 | NR | One OAT patients requiring admission and transfusion. | Patients with an INRlower than 2.2 were treated with no additionalprecautions. For patients with an INR of 2.2 to 4.0, further management including irrigation of the socket, placement of an oxidized cellulose absorbable hemostatic agent, and closure by suturing. |  | Patients taking an anticoagulant with an INR lower than 2.2 had a similar risk of bleeding as control patients. The risk was approximately 1 in 40 in those with an INR of 2.2 to 3, whereasthe risk in patients with an INR higher than 3 was approximately 1 in 11. |
| Gómez-Moreno G 2016b | Slight bleeding: slight oozing from the wound incision controlled with compressive gauze only; moderate bleeding: large clots disrupting the surgical area and requiring additional hemostatic measures; severe bleeding, requiring major medical management. | No | Non-absorbable sutures, and patients were given gauzes infused with tranexamic acid 5% to bite on for 30-60 min. |  | Dental implant surgery in patients taking dabigatran can be safely performed if 12 h have passed since the last dose, and local haemostatic measures are applied. |
| Gómez-Moreno G 2016a | Slight bleeding: slight oozing from the wound incision controlled with compressive gauze only; moderate bleeding: large clots disrupting the surgical area and requiring additional hemostatic measures; severe bleeding, requiring major medical management. | No | Non-absorbable sutures, gauze infused with tranexamic acid 5%, bite on for 30–60 min. |  | Dental implant surgery in patients taking the rivaroxaban can be safely performed applying local haemostatic measures, without modification or interruption of medication. |
| Clemm R 2016 | Low: minimal blood flow from the incision, controllable via local wound compression; Moderate: associated with the presence of blood clots in the surgical field requiring additional local hemostatic measures; Severe: artery bleeding, requiring additional hemostatic measures. | One OAT patients was hospitalized due to the severity of the bleeding as a precautionary measure. | Suturing and local wound compression with tranexamic acid gauze or operative wound revision with electrocoagulation |  | Anticoagulation therapy should be continued in patients undergoing implant surgery. Use the most minimally invasive approach to reduce postoperative risks and apply local haemostatic measures in terms of bleeding complications. |
| Bajkin BV 2015 | the bleeding continued more than 12 hours, the patient had to call the surgeon or return to the dental practice or emergency department; the bleeding resolved with a large hematoma or ecchymosis within the oral soft tissues, or the patient required a blood transfusion | No | Absorbable collagen or gelatin sponges and oxidized regenerate d cellulose as local haemostatic agents, along with wound suturing |  | Dental extractions and extensive oral surgical procedures in patients who are therapeutically anticoagulated, can be performed safely without interruption or modification of the therapy |
| Broekema FI 2014 | Severe bleeding: patient came to the hospital because the bleeding could not be stopped at home; mild bleeding: any postoperative bleeding at home that was stopped by compression with gauze | No | Patients were given standard postoperative care, and those taking vitamin K antagonists used tranexamic acid mouthwash postoperatively. |  | Dentoalveolar surgery is safe in patients treated with anticoagulants provided that the conditions described in the Academic Centre for Dentistry Amsterdam (ACTA) guidelines are met. |
| Eichhorn W 2012 | NR | No | Local haemostasis was routinely (80%) performed, with collagen fleece, local flap, and acrylic splint. |  | Oral surgical procedures can be safely performed without altering oral anticoagulant treatments. Local haemostasis seems to be sufficient to prevent postoperative bleeding. |
| Karslı ED 2011 | NR | No | The extraction socket was packed with oxycellulose dressing and sutured with 3.0 silk sutures. The patient was instructed to bite on a gauze swab for 1 hour. |  | With the aid of local haemostatic agents, dental extractions in patients receiving warfarin (INR<4) could be performed without a significant risk of bleeding and without altering their anticoagulant regimens. |
| Bacci C 2011 | mild bleeding: minor oozing from the wound incision controlled with compressive gauze only; moderate bleeding: associated with the presence of large clots continuously disrupting the surgical area and requiring additional local haemostatic measures; severe bleeding,: requiring further medical control of coagulation. | No | Non-resorbable sutures and compressive gauzes soaked with tranexamic acid |  | Dental implant surgery in anticoagulated patients can be safely performed on an outpatient basis with the simple application of local haemostatic measures. |
| Bacci C 2010 | NR | No | Local haemostatic measures (i.e., fibrin sponges, silk sutures and gauzes saturated with tranexamic acid) |  | Dental extractions can be performed easily and safely in anticoagulated outpatients without any modification of their ongoing anticoagulant therapies, thus minimizing costs and reducing patient discomfort. |
| Zanon E 2003 | NR | No | Local haemostatic measures (i.e., fibrin sponge, silk suture , a gauze saturated with tranexamic acid) |  | Dental extraction in anticoagulated patients can be safely performed on an outpatient basis, with cost reductions and minor patient discomfort. |
| Campbell JH 2000 | NR | No | NR |  | Many patients can safely undergo routine outpatient oral surgical procedures without alteration of their regular therapeutic anticoagulation regimen |

NR=not reported.
